# Prioritizing preferred traits in the yam value chain in Nigeria: a gender situation analysis

**DOI:** 10.3389/fsoc.2023.1232626

**Published:** 2023-11-13

**Authors:** Benjamin Okoye, Miriam Ofoeze, Mercy Ejechi, Samuel Onwuka, Solomon Nwafor, Nnaemeka Onyemauwa, Blessing Ukeje, Chinwe Eluagu, Jude Obidiegwu, Olamide Olaosebikan, Tessy Madu

**Affiliations:** ^1^National Root Crops Research Institute, Umudike, Nigeria; ^2^International Institute of Tropical Agriculture, Ibadan, Nigeria

**Keywords:** yam, gender team, plant breeding, trait preferences, production, processing, consumers

## Abstract

This study describes what did and did not work in the prioritization of preferred traits within the value chain of yam and associated food products (boiled and pounded yam) in Nigeria. Demand-led breeding protocols have enhanced participatory methods along gender lines to increase the clarity of information on the yam traits preferred by farmers and other end users. Drawing on the experience of the cross-cutting gender team at the National Root Crops Research Institute (NRCRI), Umudike, and partners, this study documents the successes and constraints in the use of gender-inclusive approaches for effective breeding. Methods in our gender studies involve critical assessment of the distinction between quantitative and qualitative research, with particular attention to measurement. Various techniques for data collection, such as interviews, observation, and archival studies, are assessed to locate their potential for constructing successful research projects. The methods used include participatory varietal selection, participatory plant breeding, focused discussions with farmer groups, value chain mapping, G+ tools, trait preferences (processing and consumption), triangulation of multi-disciplinary datasets, and social survey research. Yam production in southeast Nigeria is dominated by men, while women are the main processors. Gendered power play, access to resources, and decision-making have been found to constrain women's participation in yam production (and in yam research). Sex disaggregation was applied within the value chain studies to capture the complementarity and differences in the perceptions of women and men. The methods used facilitated the development and release to farmers in 2023 of three improved yam varieties with consumer-preferred characteristics such as high yield, high dry matter content, white tubers, and good boiling and pounding capability. The success stories also show that effective communication and cooperation within the gender cross-cutting team and farmer groups are important for better results. When gender specialists, food scientists, and breeders work together, innovations are created, challenges are overcome, and information is shared.

## Introduction

### Why yam breeders in Nigeria pay attention to gender

Nigeria is a multi-ethnic society; the different regions have unique cultures that influence the type of yam cultivated and the foods that consumers value (Obidiegwu and Akpabio, 2017). Breeders at the National Root Crops Research Institute (NRCRI) have been working on breeding yams that meet cultivation and use requirements. The NRCRI, in partnership with collaborators, released 35 yam varieties (Appendix 1) in Nigeria from 2001 to 2023. Roots and tuber crops are crucial in Nigeria, yams in particular, but demand for these crops is shaped by their short shelf life and by the many kinds of foods that households prepare using them. In Nigeria, yam is widely grown in regions where women contribute more farm labor, managing the use of the crop and adding value to it (Rahman, 2006).

Nigeria is the world's top yam producer, harvesting ~50 million metric tons per year (FAO, 2020), followed by Ghana (85.3 million tons), Côte d'Ivoire (77 million tons), and Benin (32 million tons). However, Nigeria's productivity ranks a distant 34th (7.9 tons per hectare), half that of nearby countries such as Ghana (8th, 18.2 tons per ha) and Benin (12th, 13.7 tons per ha). The average productivity per farm tends to be lower in countries where women make up more of the agricultural labor force than men (Udry, 1996). In several West African cultures, wealth is controlled by the man who serves as the head of the household, and in the past, yam was the ultimate form of wealth, as it was the major crop and agriculture was the main form of business (Obidiegwu and Akpabio, 2017). Hired labor in many areas of the world is performed by men, while most farm-based family labor is carried out by women and children (Shaw, 2004). Yams are not always just a women's crop or a men's crop, and their perceived gender affiliation varies widely by region and ethnic group (Cook et al., 2009). Among the Igbo people of southeast Nigeria, yam is reported to be a men's crop (Ezumah and Domenico, 1995), although Madu et al. (2021e) found evidence to the contrary. There are some areas where women play important roles in yam production, particularly water yam (probably because less value is attached to this). The present study aimed to highlight the lessons learned based on the methodologies used to prioritize preferred traits within the yam value chain in Nigeria along gender lines. The article is structured as follows: the context is first presented, followed by analyses, methods, and approaches, breeding outcomes and impacts, and discussion (good practices and lessons learned).

### Context

Such an old and important crop as the yam is surrounded by various myths, some with regional variations. Some of these myths claim that yam is not cultivated by women and cannot be harvested before the associated celebration time, otherwise it will affect the next year's harvest. In southeast Nigeria, yam is associated with a god called Ahajoku who is responsible for bumper harvests and must be appeased (but only by men). Women are sometimes allowed to cultivate other species that are considered less important, such as *Dioscorea bulbifera* or the water yam (*Dioscorea alata*), rather than the favored white yam (*Dioscorea rotundata*) or yellow yam (*Dioscorea cayennensis*). In Nigeria, yam is cultivated during a particular season, which affects the breeding program, as the output will be poor if the planting date is altered; therefore, yam is a one-season crop, unlike cassava. In the riverine areas, the yam planting season is from December to January, while elsewhere it is in April and May. Gender shapes the meaning, value, and prestige assigned to tasks (Wharton, 2011). The Igbo culture, for instance, defines yam as a male crop, and society confers prestige to male yam farmers. Higher valuation of men's work or roles is a reflection of male dominance, which is strategically aimed at granting men preferential access to opportunities and placing greater value on them as a means of exerting authority or control over others (Friedl, 1975, cited in Ubelejit-Nte and Erondu, 2022).

In Nigeria, women are mainly responsible for preparing yams to eat (Cook et al., 2009; Madu et al., 2018, 2021d). Fresh yams can be boiled, pounded, roasted, fried, etc. Boiled yam is an important food that can be eaten at all meals and as a snack. Pounded yam, a beloved food in much of Nigeria, is a glutinous dough that is mainly processed by women, who peel, boil, pound, and knead the tubers (Otegbayo, 2018). Boiled yam pieces are prepared (usually by women) by peeling, washing, and slicing the yam into pieces before boiling or steaming them (Otegbayo et al., 2005). In many places, men and women eat boiled and pounded yam approximately equally often, but this is not always the case in places where yam products are diversified (Nweke et al., 2013). For example, in Benue and Ebonyi States, Nigeria, men are more likely to eat pounded yam (Madu et al., 2018). In southwest Nigeria, tribes such as the Ijesha in Osun, Ekiti, and Ondo States have strong preferences for yam, while the Ijebus make a food called *ikokore* from the water yam. According to Obidiegwu and Akpabio (2017), yam ownership and cultivation are linked to gender and class, emphasizing male achievement and social prestige. People in the upper-income group tend to eat yams more often than the lower-income group (Nweke et al., 2013).

Barlagne et al. (2016) emphasized the dearth of knowledge of consumers' preferences regarding yam traits and product quality, despite its high nutritional value (Bradbury and Holloway, 1988) and contribution to calorie intake in West Africa (Asiedu and Sartie, 2010). Madu et al. (2021d) also noted a gap in knowledge of preferences in relation to root, tuber, and banana (RTB) crops among different user groups, e.g., food processors, retailers, and consumers, because breeding programs have historically focused on yield and other agronomic traits at the expense of post-harvest and consumer preferences. Additionally, descriptions of product traits are often oversimplified and too short, omitting information on the optimal range of an attribute that users need. Moreover, little or nothing is known about how gender relations and norms interact with preferred characteristics in relation to particular yam varieties and value chains (producers, processors, consumers, etc.).

## Analysis

### Research generated

In a bid to improve fragmented and weak crop breeding, the AfricaYam project, funded by the Bill and Melinda Gates Foundation (BMGF), began AfricaYam Phase I in October 2014, creating active yam breeding programs with faster and more precise methods for developing yam varieties that combine high and stable yield with good tuber qualities. Phase I ended in August 2020. Phase II focused on modernizing the yam breeding programs in West Africa for more efficient development of consumer-preferred varieties with higher yield, greater resistance to pests and diseases, and improved food quality following well-defined, gender-responsive product profiles.

The institutional actors driving this change are the International Institute of Tropical Agriculture (IITA); the National Root Crops Research Institute, Umudike, Nigeria; Ebonyi State University, Abakiliki, Nigeria; two research institutes under the Council for Scientific and Industrial Research (CSIR) in Ghana (the Crops Research Institute in Kumasi and the Savanna Agricultural Research Institute in Tamale); the Centre National de Recherche Agronomique (CNRA) in Côte d'Ivoire; and l'Université d'Abomey-Calavi (UAC), Dassa, Benin. In recognition of the increasing popularity of yams in East and Central Africa, the National Crops Resources Research Institute (NACRRI) in Kampala, Uganda, also joined the program. Breeding has become a multidisciplinary effort involving breeders, geneticists, food scientists, agronomists, social scientists, and end users. Research organizations outside the sub-region also play major roles in the project: the Centre de Coopération Internationale en Recherche Agronomique pour le Développement (CIRAD), Montpellier, France; the Iwate Biotechnology Research Center (IBRC), Japan; the James Hutton Institute (JHI), UK; the Japan International Research Center for Agricultural Sciences (JIRCAS), Japan; and the Boyce Thompson Institute for Plant Research (BTI), Cornell University, USA.

The beneficiaries of this intervention include farmers producing seed yam and ware yam; farmers and traders who store and wholesale seed yam and ware yam; farmers and specialist processors who transform fresh yam tubers into dry chips and yam foods; retailers of yam tubers, products, or prepared food; processors who buy dry chips from farmers and mill flour; transporters; exporters; and rural and urban consumers. The rural poor dominate yam production on small farms. Women conduct the retail trade and food processing. AfricaYam pays special attention to the rural poor and female producers and processors. The impact on traders and exporters will boost demand for yam products and enhance the livelihoods of producers and processors. Poor rural and urban consumers will benefit from reduced yam product costs for longer portions of the year, leading to improved wealth and health. Higher yields will result in more judicious use of farmland and less need to clear virgin lands. Increased income will fund farmers' capacity to invest in improved soil, water, and weed management technologies.

### How attention to gender has influenced yam breeding in Nigeria

Attention to gender in relation to breeding programs was triggered by the Breeding Program Assessment Tool (BPAT) in 2021, which recommends using a product profile to consider user preferences. The BPAT, developed with the support of the BMGF, facilitates a structured review of key technical, capacity, and management components of plant breeding programs to help design improvements that increase their efficiency and achieve higher rates of genetic gain. To modernize standard operating protocols and best practices, the BPAT recommends the more systematic use of product profiles, based on market intelligence and stakeholder consultations, to ensure that new varieties are designed to meet the preferences of women and men farmers, consumers, traders, processors, and other value chain actors (EIB, 2021). The design of new crop management systems may also be necessary, including the integration of consumers' preferences for more sustainable food systems (Selfa et al., 2008; Rastoin and Ghersi, 2010; Tsolakis et al., 2014; Barlagne et al., 2016). In the past decade, the breeding team at NRCRI has adopted the novel idea of product profiling, disaggregated by gender. Training funded by several projects (RTBfoods, NextGen, etc.) has taught researchers how to use these product profiles as a tool.

A multidisciplinary team of breeders, agronomists, pathologists, food scientists, social scientists, and gender specialists was assembled at the start of the RTBfoods project (targeting boiled yam, pounded yam, *fufu*, and *gari*). From 2017 to 2022, research findings on consumer preferences were used to develop a breeding framework to increase the efficacy of selection and adoption of improved root and tuber crop (RTC) varieties in Africa. A key factor in the success of the breeding program was the integration of knowledge contributions from all participating disciplines to enhance demand-led breeding. This was not the case in the past, which was a contributing factor in the non-adoption of some varieties. The findings were so compelling that this multidisciplinary team transformed into a gender team, which was persuaded of the necessity of considering gender to ensure that breeders could take users' wishes into account and create types that farmers would want to cultivate. The deployment of yam varieties with consumer-preferred traits incorporated into the product profiles is expected to trigger wide-scale adoption, enhancing household food availability, income, and nutrition security while improving the wellbeing of different value chain actors.

### Methods and approaches

Gender-inclusive yam studies have benefited from lessons learned in the NextGen cassava breeding project, and much later in the RTBfoods project, which captured both yam (boiled and pounded) and cassava (*gari* and *fufu*) product profiles. Robust gender-inclusive studies for yam started in 2017–2018, continuing for 5 years under the method initiated by RTBfoods (Table 1). Before RTBfoods, social scientists worked occasionally with AfricaYam breeders, although they were not considered part of the team; they held benchmark and rapid appraisals of what men and women said about certain traits, focusing on consumers, with no gender disaggregation. Outcomes from gender-inclusive studies of cassava triggered activities relating to other root and tuber crops at the NRCRI. In previous years, the gender stereotyping of yam as a male crop has constrained the inclusion of gender considerations in breeding, probably exacerbated by village meetings in which women would defer to the men in replying to questions from social scientists.

**Table 1:** Timeline of the methods used to incorporate gender-based trait preferences into yam variety development.

**Steps**	**Data gathering**	**Year**	**Team**
1. Scope of the study and gaps in research	Desktop research, workshops, and meetings to identify gaps and constraints acting as barriers to variety adoption among end users. A cross-cutting team was identified to aid the breeder's selection of prioritized traits to enhance targeted demand breeding.	2018	Gender specialist, economist, food scientist, extension specialist, and breeder.
2. Gendered food mapping: understanding the drivers of trait preferences	Participants in the value chain (producers, processors, marketers, and consumers) were identified from a gendered perspective. The multiple uses and products of yam and the possible trade-offs between uses were described. The quality characteristics and descriptors were identified by stakeholder group (e.g., producers and processors) and demand segments (e.g., rural consumers). An understanding of how gender influences preferences and the prioritization of characteristics was developed.	2018	Gender specialist, market economist, and food scientist.
3. Participatory processing diagnosis	Multiple yam varieties, including a local check, were used. Qualitative and quantitative data were elicited from four product champions to corroborate the outcomes of the gendered food mapping step.	2018	Gender specialist, market economist, food scientist, and extension specialist.
4. Consumer testing	Products from the processing diagnosis were assessed among larger groups (including men and women) in urban and rural areas to understand the consumers' demands in terms of the quality characteristics of boiled and pounded yam and to provide a clear visual mapping of the most-liked products, associated with high-quality characteristics and high overall liking scores, and of the least-liked products.	2019	Gender specialist, market economist, food scientist, breeder, and extension specialist.
5. Triangulation	Prioritized trait profiles from steps 2, 3, and 4 were triangulated to improve user acceptability of the produce (fresh yam), raw material (peeled yam), and ready-to-eat product (boiled and pounded yam).	2021	Same as above.
6. G+ tool	The G+ tool was applied at the following stages: produce (fresh yam), processing (peeled yam), and consumption (boiled and pounded yam). Weights were assigned, with intensity representing positive or negative impacts (balancing economic and non-economic drivers), thereby prioritizing trait preferences.	2022	Gender specialist, market economist, food scientist, and extension specialist.
7. Variety release	Product advancement meetings were organized to discuss and screen genotypes that should be advanced and released. Three new varieties were released in 2023 with consumer-preferred traits, such as high yield, high dry matter, flour production, and good boiling and pounding capabilities. These varieties were UMUDa35-Delight (*D. alata*, water yam), UMUDr33-Blessing, and UMUDr34-Sunshine (*D. rotundata*, white yam; Africa Yam Team, 2023).	2023	Breeders, food scientists, extension specialist, pathologist, seed system specialists, and gender specialists.

From 2018 to 2022, the team followed the steps of the RTBfoods method for integrating gender into product profiles for boiled and pounded yam (Forsythe et al., 2021; see Table 1). In Step 1, the scope of the study and the gaps in research were set, i.e., the state of knowledge (SOK) for boiled and pounded yam was established (Madu et al., 2018; Otegbayo, 2018). In Step 2, a set of ranked quality characteristics was elicited from users who played different roles in the value chain, and an in-depth social context on boiled yam (Madu et al., 2021d) and pounded yam (Otegbayo et al., 2021) was established. In Step 3, in-depth research was conducted with experienced processors to identify more quality characteristics of boiled and pounded yam (Madu et al., 2021a). The processors know yam, and they understand what the market and consumers expect in the final products. Under this method, it was found that the processors (almost all women) prepare the boiled and pounded yam in their local fashion. In one location in Anambra State, the women rejected our way of cutting the yam and presenting it to the male consumers, explaining that the men would not eat yam served our way.

Based on the results of Step 3, the team developed questionnaires for use in Step 4, which yielded robust data on preferences for the final product among a diverse set of consumers (Otegbayo et al., 2020; Madu et al., 2021b,c).

The results obtained in all the steps were then triangulated to identify priorities for the food product profile. The integrated method enables a deep understanding of quality characteristics, translating tacit knowledge into data that can be further investigated by scientists (for a discussion of tacit knowledge, see Polanyi, 1966, cited in Forsythe et al., 2021). The team also used laboratory-based sensory evaluation to corroborate the results of Steps 3 and 4, using scientific and industry-standard methods, to support breeding and product development work.

The Gender Plus (G+) tools are designed to help gender researchers and breeders to make joint, evidence-based decisions about the significance of gender differences and trade-offs beyond yield, disease resistance, tolerance of environmental stressors, and other agronomic traits (Forsythe et al., 2021; Polar et al., 2022). The team applied the G+ tools to product profiles to describe the characteristics that can determine whether or not a variety is likely to be grown or eaten as boiled or pounded yam. The use of these methods led to positive results discussed in several multidisciplinary product profile meetings, including the varietal release committee, which informed the release and adoption of new clones bred at NRCRI.

A range of methods (Table 2) were used along the whole value chain, from pre-harvest (PVS, PPB, etc.) to post-harvest (processing diagnostics, consumption trait preferences, G+ tools, social survey research, etc.). The yam varieties used for these studies were harvested as part of the AfricaYam field trials.

**Table 2:** Methods and approaches in yam variety development.

**Activities**	**Description**
Participatory varietal selection (PVS)	From 2017 to 2022, the NRCRI gender team participated in the RTBfoods and African Yam projects, employing methods involving farmers to enhance demand-led breeding. Yam tubers were selected to represent good and bad yams after a pilot sensory (taste test) evaluation with four women processors. Qualitative and quantitative information was acquired at each processing step (with raw tubers and the boiled and pounded yam), indicating how gender influences preferences and prioritization of produce and product characteristics (Madu et al., 2021b,c,d; Otegbayo et al., 2021).
Participatory plant breeding (PPB)	Analyses of data from PVS (see above) formed part of the inputs to enhance PPB.
Citizen science with mass volunteer participation	A well-structured questionnaire was used, sampling producers, processors, and yam consumers. Key informant interviews (KIIs) with women market leaders, focus group discussions (FGDs) with men and women, individual interviews (IIs), and market interviews (MIs) captured information on boiled and pounded yam. The participants signed consent forms.
Experimenting farmers organized in groups contribute to the breeding program	Women leaders (market leaders, opinion leaders, and local chairpersons of social or faith-based groups) participated as key informants. Because they command credibility in their communities, they were able to mobilize and convince other women's groups to participate in the surveys on trait preferences for targeted breeding.
Evaluation by farmers (comparisons) and selection of materials	Pair-wise ranking was adopted to enable farmers to select preferred yam varieties at various stages (fresh, peeled, boiled, and pounded) following Forsythe et al. (2021). At the production stage, more men were consulted because they dominated yam farming, while more women participated in the processing and end-product stages.
Social survey research	Sociodemographic data were gathered from the participants and interviewees.
Value chain analysis or mapping	Madu et al. (2021e) mapped the value chain for yam in the southeast region of Nigeria, including rural–urban trade flow, proportion consumed and processed, and value chain actors (men, women, youths, etc.). Nweke et al. (2013) also mapped the value chain for yam in Nigeria.
Study of trait preferences	The AfricaYam project is mainly focused on varietal creation, and the RTBfoods project focuses on the quality of RTB-based processed foods (Lebot et al., 2022). The NRCRI gender team (a cross-cutting team) participated in the RTBfoods project to complement the objectives of the AfricaYam Project and to identify quality characteristics of boiled and pounded yam for different user groups using a five-step method (Forsythe et al., 2021). Participants (disaggregated by gender and rural vs. urban) described high-quality food products from a list of sensory, processing, and agronomic characteristics.
Use of G+ tools for consumer or product profile assessments	The G+ tool (Polar et al., 2022) was used to validate and synthesize the trait preferences stated by actors on the yam value chain to prioritize breeding (Madu et al., 2022).

We organized our studies so that everyone on the gender team worked within their area of expertise. Some researchers (gender specialists and extension specialists) conducted FGDs in which farmers were separated into groups by gender. Other researchers (economists, food scientists, enumerators, etc.) held individual, key informant, and market interviews. In Ebonyi State, for example, various dialects of the Igbo language posed a communication barrier for our enumerators, but that was taken care of by the guide interpreter whom we employed for the studies. For example, in the Ezzamgbo community, one farmer described the taste and feel of a particular yam variety in the local dialect as *ona afia na onu*, which means that the yam draws very well. Guides and extension agents who understand the local dialects were able to interpret what the farmers were saying. Some of the words used in the study area were difficult to understand because they were borrowed from the languages of neighboring tribes. Some of the local dialects included sounds not found in the standard Igbo repertoire of 36 phonemes.

However, the diverse nature of the team did come with challenges when the members emphasized their own disciplines. For example, the food scientist leading the processing diagnosis and laboratory tests and the social scientist conducting consumer studies demanded yams for cooking and tasting, while the breeder and agronomist protested that this would use up the yams needed to plant the next year's trial. Unlike cassava, the yam tuber is both food and seed. This challenge almost caused the gender team to lose focus, but assertive leadership helped all the players to understand that we needed each other. During consumer studies, the breeders did not understand that we were dealing with human beings of diverse personalities, natures, and ages. The breeders were always concerned about moving to the next field trial in different locations or regions. This concern was mitigated by getting them involved in consumer studies so they could appreciate the process, which went a long way in dealing with the problem. The most effective solution was bringing all actors to the field to witness the process firsthand. This enabled us to appreciate each other's roles and attempt to accommodate one another. Meetings and presentations for sharing updates and expectations were also effective.

### Breeding outcomes and impacts

Our work shows that yams are still in high demand in Nigeria. However, 68% of those who cultivate yam as a primary livelihood belong to the poorest income group (Agbaje et al., 2005). Increased yam improvement research could reduce poverty. The gender team at NRCRI used a holistic approach with various tools (G+ tools, triangulation, ranking, etc.) to facilitate the adoption of preferred varieties.

The gender team conducted a baseline survey (Table 1, Step 2) using social survey research (Table 2), e.g., focus group discussions, key informant interviews, individual interviews, and market interviews. We documented the key priority traits desired by farmers, consumers, and marketers and found that there were similarities and differences between different sections of the country (Otegbayo et al., 2020). In addition, quality traits (study of trait preferences, Table 2) were found to vary by region; in the west of Nigeria, a food called *amala* is made from dried yam ground into flour. Drying the yams makes them dark, so the color of the tubers does not matter. However, in southeast Nigeria, white tubers were found to be important for making pounded and boiled yams. These traits were also emphasized within the triangulation and G+ tools (Table 2). The product chain includes production, usually by men assisted by women and youths on male-owned farms. Adult men usually harvest and transport the yams, while men and women sell them. Marketers and consumers include hoteliers, households, food vendors, schools, and hospitals (Madu et al., 2018). In the southeast and southwest regions, consumers were found to have preferences for certain yam traits at various stages: (1) agronomic and postharvest, (2) in processing, and (3) as prepared foods (Otegbayo et al., 2021). Men, who produce yams, emphasize yield and color, while women processers pay attention to texture, taste, ease of peeling, aroma, mealiness, poundability, and color.

In collaboration with the gender team, the agronomic team selected yam farmers to take part in the PVS on-farm trial (Table 2). Since yam is a male-dominated crop, with myths enshrining its virility, it was considered that the presence of many women could disrupt the PVS. Some communities were skeptical that we wanted to include equal numbers of women and men in trials and consumer studies. Taking that as a lesson, we decided to take it one step at a time; women sometimes opted out of the study, saying that they were not yam farmers, but they can help the men in some activities such as weeding and packing harvested yams into the barn. Women are allowed to participate in yam production if they are past reproductive age or if they are widows (and household heads). Young men and women have no rights to produce yam; they only help the men, which meant that young people were excluded from on-farm trials. However, in the course of preference ranking of varieties by farmers, we used sex disaggregation to determine the preferred varieties or trait preferences. Our holistic approach considered production, marketing, processing, and sensory characteristics (taste tests).

Consumer testing (Table 1, Step 4) in urban, semi-urban, and rural settings revealed preferred traits for boiled and pounded yam, generating positive results that led to the release in 2023 of three promising new varieties bred by the NRCRI in collaboration with the IITA: UMUDa35-Delight, UMUDr33-Blessing, and UMUDr34-Sunshine. These remarkable varieties are tasty, high-yielding, high in dry matter, well-suited for making flour, boiling, and pounding, and resistant to anthracnose disease (Table 1, variety release; Africa Yam Team, 2023). This long-term commitment to consulting farmers' opinions or involving them directly in selection can produce notable improvements in adoption rates, and released varieties will be more strongly appreciated by farmers, especially women in Africa (Witcombe et al., 2005; Njuguna et al., 2016; Weltzien et al., 2019; Polar et al., 2021).

## Discussion

The study investigated the prioritization of traits within the yam value chain in Nigeria along gender lines, from planting to plate. Surveys and on-farm trials used methods that involved critical assessment (both quantitative and qualitative), with particular attention to measurement. Various data collection and fact-finding instruments were used, such as individual, market, and key informant interviews, observations, and FGDs, to enhance demand-driven research and prioritize traits to aid breeding. The data elicited were synthesized using various methods, particularly the G+ tool, which helped in prioritization and in defining trade-offs between preferred traits. The cross-cutting gender team facilitated successes, good practices, and lessons learned, culminating in the release of three improved varieties with some of the traits prioritized by consumers. This approach is expected to increase the adoption of the new yam varieties and can also serve as a guide for future research studies.

### Good practices

#### Markets and consumers

Participants were gender-disaggregated to include each node of the value chain (Table 1, step 2). These nodes include the direct players (producers, processors, and consumers) and the indirect players who facilitate the chain (transporters, loaders, input dealers, and credit agencies). All these actors had a say in describing the yam qualities that would meet demand, leading to enhanced livelihoods.

#### Breeding objectives

Going forward, all projects now have a gender component that will incorporate a benchmark study and a rapid appraisal as a guide for breeders. Incorporating consumers' preferences avoids the disadoption of released varieties. Working together in the field, as well as analyzing and triangulating agronomic, processing, and consumption data, informs and guides breeders.

#### Methods for evaluating varieties

Gender-disaggregated multidisciplinary studies have begun to be incorporated into the early stages of variety design to enhance the consideration of gender in prioritization of preferred traits.

#### Seed multiplication and dissemination

The use of certified seed (cassava, yam, and sweet potato) by companies such as Umudike Seeds Ltd., village seed entrepreneurs (VSEs), aeroponics, and various research institutes helps to ascertain the quality of the seeds being disseminated and keep track of them. VSEs help in the rapid dissemination of planting materials; they comprise men and women who serve as outgrowers and maintain quality control in the production of seed.

### Lessons learned

#### Breeding strategy

The bottom–top approach, with a mix of methods, promoted a better understanding of the breeding protocol and of how to develop demand-led, gender-intentional product profiles.

#### Criteria for evaluating the importance of traits

Pair-wise ranking, hedonic scales, scores, percentages, and ranks were used to rate the importance of choice traits (Table 1, Steps 2, 3, and 4). We needed to be careful to avoid confusing the use of ranks (1 through 5), where 1 was the most preferred, with mean scores (where 5 represented the most preferred), but we were successful in this. When we evaluated multiple traits, some were tied with each other for the same rank. The G+ tool helped to disentangle the importance of tied traits.

#### Relative importance or weight given to different traits

In future yam studies, economic values will be assigned to traits to prioritize actors' preferences for those traits (Balogun et al., 2022). This method changes the traditional economic approach to ranking tools, balances economic and non-economic drivers, and quantifies the views of participants by gender and social strata.

#### Prioritization of traits in breeding

This involves the use of a multidisciplinary approach from the start of breeding to select the traits that end users prefer and to involve these actors (gender disaggregated). Breeding for end users facilitates the release and adoption of new varieties. Holistic information should be provided by all stakeholders. Women should be encouraged to become involved in the different stages, not only in cultivation, which is dominated by men.

#### Choice of materials to advance to the next stage of breeding

High- and medium-throughput methods like near-infrared spectroscopy should be developed and used. Consumer-preferred traits should be correlated with the biophysical properties identified in the lab to obtain a threshold for selection (RTBfoods). High-throughput methods save time and money when making breeding decisions, so that promising clones will not be dropped but non-promising ones will be. This method has already been developed for rapid characterization of the color and texture of gari-eba.

## Data availability statement

Publicly available datasets were analyzed in this study. This data can be found at: https://agritrop.cirad.fr/602044/, https://agritrop.cirad.fr/602099/, https://ifst.onlinelibrary.wiley.com/doi/full/10.1111/ijfs.14770, https://www.researchgate.net/publication/351706422_State_Of_Knowledge_Report_Synthesis_Report_for_Yam_in_Nigeria, https://mel.cgiar.org/reporting/download/report_file_id/25482, https://agritrop.cirad.fr/602029/, https://agritrop.cirad.fr/602029/1/RTBfoods_Participatory%20processing%20diagnosis_Boiled%20yam_Nigeria.pdf, https://mel.cgiar.org/reporting/download/report_file_id/25467.

## Ethics statement

The studies involving humans were approved by the National Root Crops Research Institute that has the mandate for Root and Tuber Crops Research in Nigeria. The studies were conducted in accordance with the local legislation and institutional requirements. The participants provided their written informed consent to participate in this study. Written informed consent was obtained from the individual(s) for the publication of any potentially identifiable images or data included in this article.

## Author contributions

All authors made significant contribution to the study, conception, study design, execution, acquisition of data, analysis, and interpretation. All authors read through the different drafts of the study, and made substantially and critical contributions to the review of the article. BO contributed to most to the study and including writing of the manuscript. All authors contributed to the article and approved the submitted version.
