# Supplementary material for: Prioritizing preferred traits in the yam value chain in Nigeria: a gender situation analysis
Source: Front Sociol. 2023 Nov 13;8:1232626. doi: 10.3389/fsoc.2023.1232626 (PMC10679750; doi:10.3389/fsoc.2023.1232626)
Supplement: Supplementary file 1 [file Table_1.docx]

**Appendix 1: List of 35 released yam varieties in Nigeria**

| **S/N** | **Variety name** | **Original name** | **Outstanding Characteristics** | **Year of release** |
| --- | --- | --- | --- | --- |
| 1 | TDR 89/02677 | TDR 89/02677 | Stable yield, very good cooking and pounding qualities, cream tuber parenchyma, 25% tuber  dry matter content. | 2001 |
| 2 | TDR 89/02565 | TDR 89/02565 | Stable yield, very good cooking and pounding qualities, cream non-oxidizing parenchyma,  35% tuber dry matter | 2001 |
| 3 | TDR 89/02461 | TDR 89/02461 | Stable yield, very good as cooking and pounding qualities, cream parenchyma, 26.7% tuber  dry matter. | 2001 |
| 4 | TDR 89/02665 | TDR 89/02665 | Stable yields very good cooking and pounding qualities, cream non-oxidizing parenchyma,  35.3% tuber dry matter | 2003 |
| 5 | TDR 89/01213 | TDR 89/01213 | cooking and pounding qualities, white non-oxidizing parenchyma, tuber dry matter-  29.8% | 2003 |
| 6 | TDR 89/01438 | TDR 89/01438 | Stable yield, very good cooking and pounding qualities, white non-oxidizing parenchyma, tuber dry matter  29.3% | 2003 |
| 7 | TDR 95/01924 | TDR 95/01924 | Stable yield, very good cooking and pounding qualities, white non-oxidizing parenchyma, tuber dry matter-  32.8% | 2003 |
| 8 | DRN 200/4/2 | DRN 200/4/2 | High yielding, pests and disease tolerant, very good for fufu, frying and  Boiling (35t/ha) | 2008 |
| 9 | TDa98/01176 | TDa98/01176 | High yielding, pests and disease tolerant, good for pounded yam, frying and boiling, suitable for both rainy and dry seasons yam  production. (26-30t/ha) | 2008 |
| 10 | TDa98/01168 | TDa98/01168 | High-yielding, pests and disease tolerant, good for pounded yam frying and boiling. (24-28t/ha) | 2008 |
| 11 | TDa98/01166 | TDa98/01166 | High yielding, pests and disease tolerant, good for pounded yam, frying and boiling, suitable for both rainy and dry seasons yam  production. (26-30t/ha) | 2008 |
| 12 | TDr 95/19158 | TDr 95/19158 | High yielding, pests and disease tolerant, very good for yam, fufu, frying and boiling. (29.4t/ha) | 2009 |
| 13 | TDr 89/02602 | TDr 89/02602 | High yielding, pests and disease tolerant, very good for yam, fufu, frying and boiling. (31.5t/ha) | 2009 |
| 14 | TDr 89/02660 | TDr 89/02660 | High yielding, pests and disease tolerant, very good for yam, fufu, frying and boiling. (31t/ha) | 2009 |
| 15 | TDa 00/00194 | TDa 00/00194 | High yielding, pests and disease tolerant, good for pounded yam, frying  and boiling. (37.5t/ha | 2009 |
| 16 | TDa 00/00104 | TDa 00/00104 | High yielding, pests and disease tolerant, good for pounded yam, frying  and boiling. (30t/ha) | 2009 |
| 17 | UMUDa-4 | TDa 00/00364 | High yielding, good for Amala, pounded yam, frying and boiling.  (33.3t/ha) | 2010 |
| 18 | UMUDr-17 | TDr 95/19177 | High-yielding under dry season yam cropping  system. (30t/ha) | 2010 |
| 19 | UMUDr-18 | TDr 89/02475 | High yielding, pests and disease tolerant, very good for yam fufu, frying  and boiling. (31t/ha) | 2010 |
| 20 | UMUDr-20 | TDr 98/00933 | Potential yield (39.8t/ha) | 2016 |
| 21 | UMUDr-21 | 99/Amo/064 | Potential yield (43.9t/ha) | 2016 |
| 22 | Obiaoturugo | Obiaoturugo | High yielding. (27.78t/ha | 2016 |
| 23 | Amola | Amola | High yielding. (21.6t/ha | 2016 |
| 24 | Hembakwasa | Hembakwasa | High yielding. (29.94t/ha | 2016 |
| 25 | Ekpe | Ekpe | Early maturing and high yielding. (23.21t/ha) | 2016 |
| 26 | Alushi | Alushi | High yielding. (27.6t/ha) | 2016 |
| 27 | UMUDa-27 (Aku abata) | TDa1100201 | Slow rate of oxidization (browning) and high dry matter content. (35t/ha | 2019 |
| 28 | UMUDa-28 (VaYam) | TDa1100316 | Non-browning after processing, excellent boiling and pounding quality. (34t/ha) | 2019 |
| 29 | UMUDr-29  (Super) | UMUDr-29  (Super) | Slow rate of oxidization (browning) and high dry matter content. High yield (22t/ha) | 2020 |
| 30 | UMUDr30 (Nagode) | TDr1000048 | Slow rate of oxidization (browning) and high dry matter content., high yield(24t/ha) | 2020 |
| 31 | UMUDa 31 (Wonder) | TDa1100432 | Slow rate of oxidization (browning) and high dry matter content, high yield, excellent boiling and pounding quality. (43t/ha) | 2020 |
| 32 | UMUDr32  (Favorite) | TDr1100497 | High dry matter, high tuber and flour yields, high starch, slow rate of oxidation, excellent sensory properties. (32.7t/ha) | 2022 |
| 33 | UMUDr33  (Blessing) | TDr1401220 | High yield (33t/ha), high dry matter (36.4%), Good boiled and pounded yam quality | 2023 |
| 34 | UMUDr34  (Sunshine) | TDr1400158 | High yield (33t/ha), high dry matter (36.4%), excellent boiled and good pounded yam quality | 2023 |
| 35 | UMUDa35  (Delight) | TDa1100374 | High yield 45.7(t/ha), high dry matter (33.3%), and high flour yield (26.4%) | 2023 |
